# A Novel Role for the SMG-1 Kinase in Lifespan and Oxidative Stress Resistance in *Caenorhabditis elegans*

**DOI:** 10.1371/journal.pone.0003354

**Published:** 2008-10-06

**Authors:** Ingrid Masse, Laurent Molin, Laurent Mouchiroud, Philippe Vanhems, Francesca Palladino, Marc Billaud, Florence Solari

**Affiliations:** 1 Laboratoire de Génétique, Signalisation et Cancer, Université Claude Bernard Lyon 1, CNRS UMR5201 Domaine Rockefeller, Lyon, France; 2 Unité d'Epidémiologie et d'Hygiène Hospitalière, Hôpital Edouard Herriot, Hospices Civils de Lyon et Laboratoire d'Epidémiologie et de Santé Publique, Université de Lyon, Université Lyon 1, CNRS, UMR 5558, Laboratoire de Biométrie et Biologie Evolutive, Lyon, France; 3 Laboratoire de Biologie Moléculaire de la Cellule, Ecole Normale Supérieure de Lyon, CNRS UMR5161, Lyon, France; Massachusetts General Hospital/Harvard Medical School, United States of America

## Abstract

The *PTEN* tumour suppressor encodes a phosphatase, and its *daf-18* orthologue in *Caenorhabditis elegans* negatively regulates the insulin/IGF-1 DAF-2 receptor pathway that influences lifespan in worms and other species. In order to identify new DAF-18 regulated pathways involved in aging, we initiated a candidate RNAi feeding screen for clones that lengthen lifespan. Here, we report that *smg-1* inactivation increases average lifespan in a *daf-18* dependent manner. Genetic analysis is consistent with SMG-1 acting at least in part in parallel to the canonical DAF-2 receptor pathway, but converging on the transcription factor DAF-16/FOXO. SMG-1 is a serine-threonine kinase which plays a conserved role in nonsense-mediated mRNA decay (NMD) in worms and mammals. In addition, human SMG-1 has also been implicated in the p53-mediated response to genotoxic stress. The effect of *smg-1* inactivation on lifespan appears to be unrelated to its NMD function, but requires the *p53* tumour suppressor orthologue *cep-1*. Furthermore, *smg-1* inactivation confers a resistance to oxidative stress in a *daf-18*-, *daf-16*- and *cep-1*-dependent manner. We propose that the role of SMG-1 in lifespan regulation is at least partly dependent on its function in oxidative stress resistance. Taken together, our results unveil a novel role for SMG-1 in lifespan regulation.

## Introduction

The insulin/IGF-1 DAF-2 receptor pathway is implicated in lifespan control in several species [1]. In *C. elegans*, DAF-2 signals to the PI3 kinase (PI3K) homolog AGE-1. AGE-1 generates phosphatidyl inositol 3, 4, 5 triphosphate, or PIP3, which is in turn responsible for the activation of the AKT-1, AKT-2 and SGK-1 serine threonine kinases in a PDK-1 dependent manner [2]–[5]. Phosphorylation of the FOXO transcription factor DAF-16 by these kinases inhibits DAF-16 function by inducing its sequestration into the cytoplasm [6]–[8]. The DAF-2 receptor pathway is downregulated by DAF-18, which is encoded by the *C. elegans* orthologue of the human tumor suppressor gene *PTEN*. Like *PTEN*, *daf-18* encodes a PIP3 phosphatase that antagonizes the activity of AGE-1 in the DAF-2 pathway to regulate lifespan [9]–[13]. As *daf-18* mutations suppress the lifespan phenotype of *daf-2* and *age-1* mutants, it has been proposed that the role of DAF-18 in lifespan regulation relies on inhibition of the insulin/IGF-1 signaling pathway (Mihaylova *et al.*, 1999).

In order to identify new DAF-18 partners, we initiated a candidate RNAi feeding screen for clones that lengthen lifespan in a *daf-18* dependent manner, focusing on potential protein kinases. We identified one clone encoding the protein kinase SMG-1. Genetic analyses strongly suggest that SMG-1 acts in parallel to DAF-2, AGE-1 and AKT-1, but requires DAF-16, to modulate lifespan. SMG-1 is conserved across species and is involved in a mechanism responsible for the degradation of premature stop codon containing mRNA, also called NMD for “nonsense mediated mRNA decay” in *C. elegans* and in mammalian cells [14]–[16]. Interestingly the role of SMG-1 in lifespan appears to be unrelated to its function in NMD, but requires the *p53 C. elegans* ortholog, *cep-1*, *daf-18,* and *daf-16*. Furthermore, our results uncover a role for SMG-1 in oxidative stress response that may be responsible for its effect on lifespan. Overall, our study unveils a novel role for SMG-1 in oxidative stress response and lifespan regulation that may be conserved in mammals.

## Results and Discussion

### *smg-1* inhibition increases average lifespan in a *daf-18* dependent manner

The screen was performed with *rrf-3 (pk1426)* single and *rrf-3 (pk1426); daf-18 (e1375)* double mutants. The *daf-18 (e1375)* mutation is a hypomorphic allele of *daf-18,* while the *rrf-3* mutant was chosen because it shows an enhanced sensitivity to RNAi feeding [17]. As expected, RNAi of genes acting in the insulin pathway, including *daf-2, age-1* and *akt-1*, resulted in a *daf-18* dependent lengthening of lifespan, validating our experimental approach.

**Figure 1:**
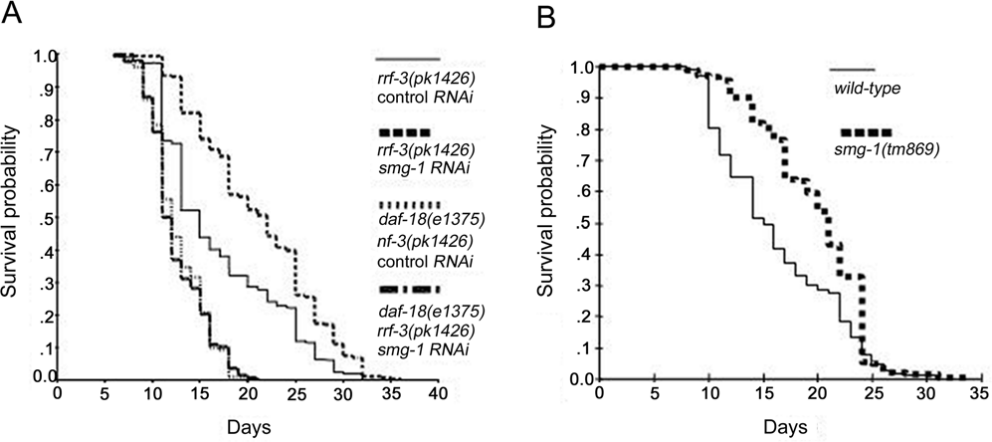
smg-1 inactivation increases mean lifespan in a *daf-18* dependent manner. (A) Survival curves of *rrf-3(pk1426)* and *daf-18(e1375); rrf-3(pk1426)* mutants fed either bacteria not expressing any dsRNA (control RNAi) or bacteria expressing dsRNA that targets *smg-1* (two independent clones gave similar results). For additional data, see Table 1. (B) Survival curves of *smg-1(tm869)* mutants and wild-type used for backcrosses (see Materials and Methods).

In addition, from the 269 hand picked clones tested (Table S1) we identified one corresponding to the *smg-1* gene. The average lifespan of the *rrf-3* strain was increased by 25% when worms were fed either one of the two non-overlapping RNAi clones for *smg-1* (C48B6.6 and C48B6.7) and the increase in lifespan was completely suppressed in *rrf-3(pk1426); daf-18(e1375)* mutants (Table 1; Figure 1A). Therefore, inhibition of *smg-1* by RNAi increases lifespan, and this effect requires DAF-18 activity.

**Table 1:** Effect of *smg-1* inactivation on adult lifespan in different genetic backgrounds.

Genotypes	RNAi	Mean lifespan+/-SE (days)	Median lifespan (days)	*P-*values against control ^a^	*P-*values against specific group	# death/# total (# trials)^i^
wild-type		16.2± 0.4	15			229/234(3)
*smg-1(tm869)*		19.8± 0.3	21	<10^−3^		187/351(3)
*rrf-3(pk1426)*	control	16.9 ± 0.2	15			701/925 (10)
	*smg-1* *	21.2 ± 0.3	22	<10^−3^		540/969 (11)
	*smg-2*	15.9± 0.4	15	0.002		158/169 (2)
	*smg-4*	18.9± 0.6	18	0.07		94/179 (2)
	*smg-5*	18.5± 0.5	18	0.074		133/182 (2)
	*smg-7*	17± 0.6	15	0.9		97/180 (2)
	*cep-1*	18.6 ± 0.5	15	0.006		246/300 (3)
	*smg-1+cep-1*	20 ± 0.5	19	<10^−3^	0.03^ b^	227/294 (3)
	*daf-2*	31.1 ± 0.6	32	<10^−3^		289/351 (4)
	*age-1*	23.6 ± 0.5	22	<10^−3^		313/396 (4)
	*daf-2+age-1*	30 ± 0.8	32	<10^−3^	0.48^ c^	160/184 (2)
	*smg-1+daf-2*	37.3 ± 0.7	39	<10^−3^	<10^-3 c^	147/177 (2)
	*smg-1+age-1*	31.1 ± 0.8	32	<10^−3^	<10^−3 d^	147/193 (2)
	*akt-1*	25.3 ± 0.6	26	<10^−3^		138/198 (2)
	*smg-1+akt-1*	29.7 ± 0.6	32	<10^−3^	<10^−3 e^	134/196 (2)
	*daf-18*	14.7 ± 0.3	14	<10^−3^		144/200 (2)
	*daf-16*	12.6 ± 0.2	12	<10^−3^		156/289 (3)
	*smg-1+daf-16*	12.3 ± 0.2	12	<10^−3^	0.49^ f^	114/199 (2)
	*daf-19*	20.9 ± 0.6	21	<10^−3^		162/288 (2)
	*smg-1+daf-19*	21.9 ± 0.7	21	<10^−3^	0.09^ b^	101/174 (2)
	*daf-19+daf-18*	14.4 ± 0.3	14	<10^−3^	0.52^ g^	134 /202(2)
	*daf- 19+daf-16*	13.4 ± 0.3	12	<10^−3^	<10^−3 h^	105/198 (2)
*daf-18(e1375); rrf-3(pk1426)*	control	12.7 ± 0.2	12	0.79		202/226 (3)
	*smg-1*	12.5 ± 0.3	12			138/197 (3)
*tax-4(p678); rrf-3(pk1426)*	control	22.6 ± 0.5	24	<10^−3^		146/201 (2)
	*smg-1*	26.5 ± 0.6	26			105/192 (2)

All experiments were carried out by RNAi feeding at 20°C (see experimental procedures). ^a-i^: *p*-values from a log rank test comparing RNAi treatment population to the vector control ^a^ or to specific groups (*smg-1* ^b^, *daf-2* ^c^, *age-1* ^d^, *akt-1* ^e^, *daf-16* ^f^, *daf-18* ^g^ or *daf-19* ^h^ RNAi). *P-*values less than 0,05 are considered statistically significant, demonstrating that the two lifespan populations are different. ^*^: results obtained with two independent RNAi feeding clones that gave similar results have been pooled.^i^ The total number of individuals scored is shown followed by the number of individuals censored due to bursting vulva, bagging, or crawling off the agar. Data obtained in individual tests are reported in Table S2.

We next tested whether the previously isolated *smg-1(r861)* null allele [18] also shows a lifespan phenotype. The average lifespan of *smg-1(r861)* mutant animals was not increased compared to wild-type animals (data not shown). Nonetheless, despite the fact that these mutants did not live longer than wild-type, we observed a delayed accumulation of the aging marker lipofuscine [19] during the first week of life of *smg-1(r861)* mutants, as observed in *smg-1* RNAi treated animals (data not shown).

Furthermore, in agreement with previously published data *smg-1(r861)* null mutants are associated with a fully penetrant protruding vulva phenotype (94%; n = 205), while this phenotype was only observed in 31% (n = 969) of *smg-1* RNAi treated animals. The majority of other *smg-1* mutants we tested behave like the *smg-1(r861)* null allele (data not shown) besides *smg-1*(*tm869)* mutants (recently isolated by the Japanese *C.elegans* knockout consortium). An exception is the *smg-1*(*tm869)* allele, which results in a protruding vulva phenotype with similar penetrance (47%, n = 351) to *smg-1* RNAi fed animals. Indeed, lifespan tests revealed that the *smg-1(tm869)* mutation increases average lifespan by more than 20% (Figure 1B and Table 1).

Overall, our results suggest that RNAi mimics a hypomorphic mutation, while complete loss of SMG-1 function is deleterious and masks a longevity phenotype.

### SMG-1 may act in parallel of the insulin/IGF-1 DAF-2 receptor

Since DAF-18 functions in the insulin/IGF-1 DAF-2 receptor pathway to regulate lifespan, we tested whether SMG-1 also acts in this signaling cascade.

**Figure 2:**
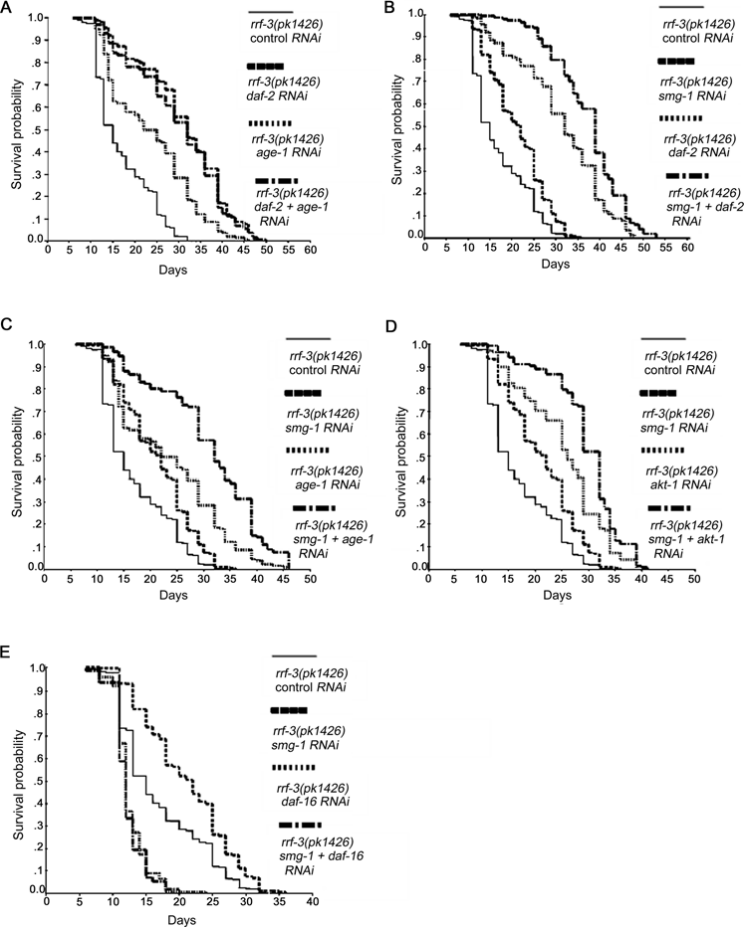
Interaction between *smg-1* and the insulin/IGF-1 signalling pathway genes for lifespan phenotype. (A) Survival curves of *rrf-3(pk1426)* mutants treated by *daf-2* and/or *age-1* RNAi. (B-E) Survival curves of *rrf-3(pk1426)* mutants treated by *daf-2* (B), *age-1* (C), *akt-1* (D) and *daf-16* (E) RNAi alone or with *smg-1* RNAi. For additional data, see Table 1.

We favored RNAi approaches to assess epistatic relationships between *smg-1* and the different components of the insulin pathway in order to analyse data in an isogenic background. RNAi of *daf-2* increased lifespan by 84% compared to control RNAi (Table 1; Figure 2B). RNAi of both *daf-2* and *smg-1* further extended the average lifespan to 120% (Table 1; Figure 2B). Similarly, the average lifespan of *age-1* RNAi and *akt-1* RNAi treated worms was further increased from 40 to 84% and from 50 to 75%, respectively, when fed with *smg-1* RNAi (Table 1; Figure 2 C,D). Conversely, the lifespan of animals treated with RNAi for both *daf-2* and *age-1,* which act in the same pathway, was not significantly different from the lifespan of *daf-2* RNAi worms alone (Table 1; Figure 2A). Therefore, *smg-1* inactivation increases lifespan independently of *daf-2*, *age-1* or *akt-1.* However, the extension of lifespan by *smg-1* RNAi was completely suppressed when *daf-16* was inactivated by RNAi (Table 1; Figure 2E). Overall, these data suggest that SMG-1 may act in a pathway parallel to DAF-2, AGE-1 and AKT-1, but requiring DAF-16 activity.

Nonetheless, because gene inactivation by RNAi mimics a hypomorphic rather than a null mutation, we cannot formally exclude that the insulin receptor pathway partially contributes to the *smg-1* effect on lifespan.

**Figure 3:**
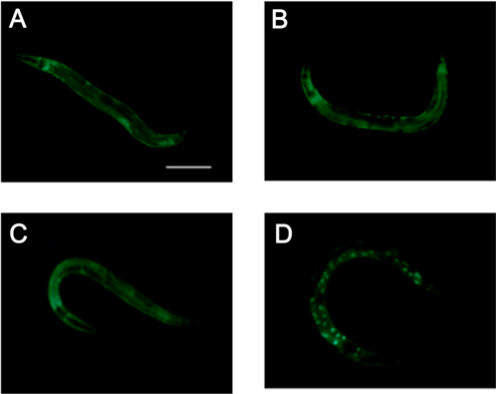
DAF-16∶∶GFP sub-cellular localization is not altered by *smg-1* inactivation. DAF-16∶∶GFP is localized both in the cytoplasm and nuclei of all tissues at all developmental stages of *rrf-3(pk1426)* (A) and wild-type (B) control animals as illustrated here for adults. Similar results were obtained for *rrf-3(pk1426) smg-1* RNAi-treated animals (C) and *smg-1(r861)* single mutants (data not shown). (D) DAF-16∶∶GFP accumulates in the nuclei of most cells in *rrf-3(pk1426)* animals treated with *daf-2* RNAi. Scale bars: 100 µm.

DAF-16 function can be modulated through its nuclear localization [6], [7], [20], [21]. To investigate whether SMG-1 controls DAF-16 sub-cellular localization, we made use of a strain carrying a *daf-16∶∶gfp* reporter construct to visualize nuclear translocation *in vivo* [6]. DAF-16∶∶GFP was localized in both the cytoplasm and the nucleus in all tissues of worms after *smg-1* inactivation by RNAi or by mutation (Figure 3), as observed in control worms. Conversely, under the same experimental conditions, *daf-2* RNAi induced DAF-16∶∶GFP nuclear accumulation (Figure 3). These results strongly suggest that SMG-1 does not regulate DAF-16 activity through its sequestration into the cytoplasm, and further support the idea that SMG-1 and DAF-2 may act in different pathways to regulate lifespan.

Nonetheless, these observations do not exclude that SMG-1 may behave as a weak enhancer of the insulin pathway, since Henderson and Johnson [6] showed that some *age-1* mutants also fail to induce nuclear re-localization of this reporter. We thus addressed the relationship between the DAF-2 pathway and SMG-1 by a third approach.

The DAF-2 pathway is also critical for controlling dauer formation. To further assess a potential functional link between SMG-1 and DAF-2 we tested the involvement of SMG-1 in dauer formation. One would predict that if the role of SMG-1 in lifespan control relies on its interaction with the DAF-2 pathway, then SMG-1 inhibition should increase dauer formation. However, we found that *smg-1* RNAi slightly increased the ability of worms to recover from larval arrest rather than enhancing dauer formation. When *daf-2(e1370)* or *daf-2(e1370); rrf-3 (pk1426)* double mutants were maintained at the semi-nonpermissive temperature 22°C [22], 78+/−7% of *smg-1* RNAi treated worms had reached the adult stage after 4 days, compared to 68+/−4.8% of worms grown on HT115 control bacteria (*P* = 0.02). These data strongly suggest that SMG-1 is not a broad positive regulator of the insulin pathway.

Overall our data support a model in which SMG-1 functions at least in part independently of DAF-2 to regulate lifespan.

### The role of SMG-1 in lifespan control does not depend on its function in NMD

*smg-1* encodes a conserved serine threonine kinase involved in nonsense mediated mRNA decay (NMD), a mechanism responsible for the degradation of mRNAs containing a premature stop codon [14]-[16].

In addition to SMG-1, six evolutionary conserved SMG proteins are also required for NMD in *C. elegans* and in mammalian cells. Genetic studies have determined that *smg* genes are regulators of the phosphorylation state of SMG-2. SMG-1, SMG-3 and SMG-4 are required for the phosphorylation of SMG-2, whereas SMG-5, SMG-6 and SMG-7 are involved in its dephosporylation [23].

**Figure 4:**
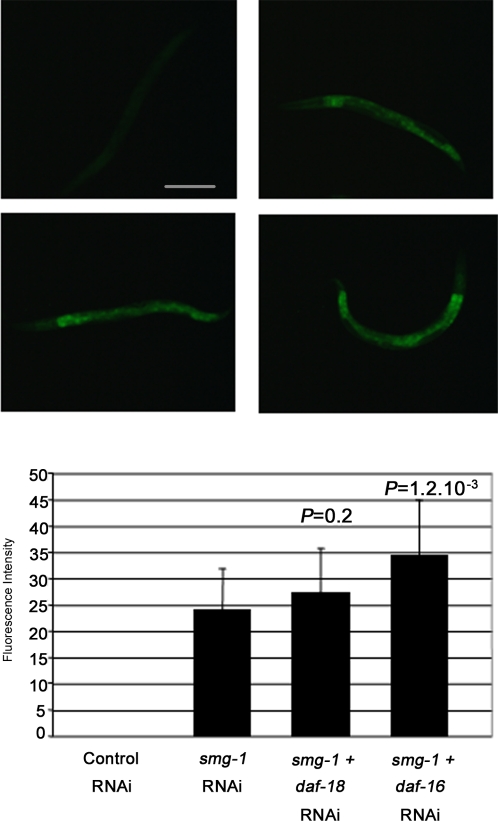
The SMG-1 dependent effect on NMD regulation is not suppressed upon DAF-18 or DAF-16 inactivation. Animals carrying the PTC transgenic reporter were fed with the different RNAi clones and scored for GFP expression (see experimental procedures). Three independent experiments were carried out and gave similar results. (A) Animals treated with the control (n = 80) lack GFP expression. Conversely, 100% of worms treated by *smg-1* RNAi (n = 80) (B), or by *smg-1* and *daf-18* RNAi (n = 60) (C), or by *smg-1* and *daf-16* RNAi (n = 50) (D) showed GFP expression. Scale bar: 200 µm. (E) GFP quantification using arbitrary units (see experimental procedure). The error bars reflect the variation between animals. *P* values correspond to comparison of average GFP intensity between the test group and worms treated with *smg-1* RNAi using Student's *t* test.

If the role of SMG-1 in lifespan relies on its function in NMD, inactivation of other *smg* genes should also have an effect on lifespan. The average lifespan of worms fed with *smg-2, smg-4*, *smg-5* or *smg-7* RNAi clones was not significantly increased (Table 1), suggesting that NMD inactivation may not be responsible for lifespan extension. To further explore this hypothesis, we assess NMD activity in *smg-1* RNAi treated worms. Longman et al. [24] developed an assay using transgenic strains expressing a GFP reporter constructs either with a natural stop codon or harboring a premature termination codon (PTC). Introduction of a PTC into the reporter induces a robust NMD response, as determined by the lack of GFP expression in transgenic worms. Conversely, *smg-2* RNAi, which abrogates NMD, restores GFP expression [24]. Under our experimental conditions, GFP expression was induced in 100% of PTC transgenic worms fed with the *smg-1* RNAi clone (Figure 4), demonstrating the effectiveness of *smg-1* RNAi feeding in NMD inhibition. *daf-18* and *daf-16* are required for the *smg-1* dependent lifespan increase. We reasoned that if NMD inhibition is responsible for the role of *smg-1* in lifespan control, inactivation of *daf-18* or *daf-16* should antagonize this function and thus impede GFP expression in *smg-1* RNAi treated PTC transgenic worms. Inactivation of either *daf-18* or *daf-16* by RNAi, which is sufficient to suppress the *smg-1* lifespan phenotype, did not reduce the number of GFP expressing worms. Furthermore, the level of GFP expression in individual worms was unaffected or increased by *daf-18* and *daf-16* RNAi, respectively (Figure 4).

Overall our results show that there is no correlation between NMD inactivation and lifespan phenotypes, thus indicating that SMG-1 functions independently of NMD to regulate lifespan.

### *p53/cep-1* is involved in *smg-1* dependent lifespan modulation

It was recently reported that human SMG-1 is functionally linked to the tumor suppressor checkpoint protein p53. hSMG-1 phosphorylates and stabilizes p53 in response to genotoxic stress induced by UV and γ irradiation [25]. In worms, the *p53* homologue *cep-1* is required for DNA damage-induced apoptosis [26], [27]. Interestingly, Arum et al. recently showed that *cep-1* mutations also increase longevity without altering DAF-16∶∶GFP nuclear localization [28]

**Figure 5:**
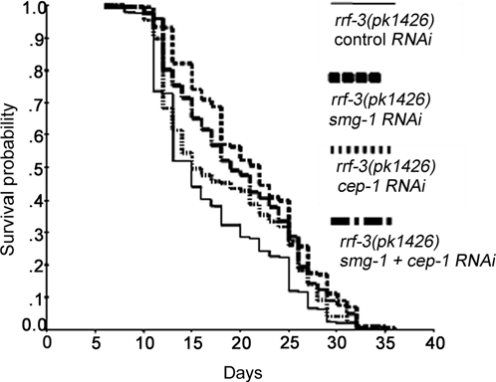
SMG-1 requires CEP-1 to regulate lifespan. Survival curves of *rrf-3(pk1426)* mutants treated by *smg-1* and/or *cep-1* RNAi. This figure uses the same data set as Table 1.

We therefore tested whether *cep-1* is involved in the regulation of lifespan by *smg-1. cep-1* RNAi partially suppressed the extension of lifespan due to *smg-1* inhibition (Table 1; Figure 5). The genetic interaction between *cep-1* and *smg-1* is specific, as *cep-1* RNAi alone did not reduce lifespan (Table 1; Figure 5). These results indicate that when *smg-1* is inactivated, *cep-1* is required to extend lifespan.

### *smg-1* inactivation confers resistance to oxidative stress

**Figure 6:**
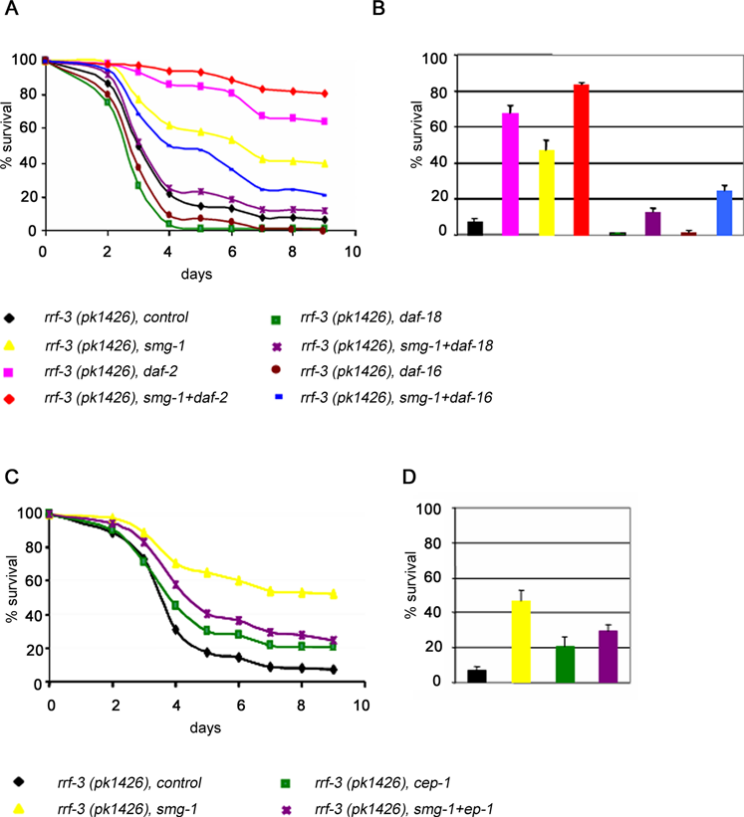
SMG-1 regulates resistance to oxidative stress in a DAF-18, DAF-16 and CEP-1 dependent manner. (A, C) Survival curves of RNAi treated animals in presence of paraquat (see materials and methods). (B,D) Histograms presented as mean ± SEM at day 7 of paraquat treatment. *smg-1(r861)* null mutants behave similarly to *smg-1* RNAi animals (data not shown). Genetic interactions for oxidative stress resistance between *smg-1* and either *daf-2*, *daf-18* or *daf-16* (A, B) and between *smg-1* and *cep-1* (C, D).

Resistance to oxidative stress is a hallmark of many longevity mutants in *C. elegans* [29]. Consistently, we observed that worms were more resistant to oxidative stress induced by paraquat when *smg-1* was inactivated by RNAi or by mutation, as 47% of worms were still alive after 7 days of paraquat treatment compared to 7% for control RNAi (Figure 6 A, B). Conversely, *daf-18* and *daf-16* RNAi inhibited the resistance of worms to oxidative stress compared to control RNAi and dramatically reduced the stress resistance induced by *smg-1* inactivation (Figure 6 A, B). These results show that SMG-1 requires DAF-16 and DAF-18 to confer oxidative stress resistance as well as to function in lifespan control. A correlation between lifespan and oxidative stress resistance phenotypes was also observed for the genetic interaction between *daf-2* and *smg-1,* as the resistance to oxidative stress of *daf-2* RNAi treated animals was further increased by *smg-1* inactivation (Figure 6 A, B; p<10^−3^).

To further explore the relationship between lifespan and resistance to oxidative stress phenotypes, we investigated the role of *cep-1/p53* in oxidative stress resistance. Under experimental conditions where *cep-1* RNAi increased lifespan (Figure 5), resistance to oxidative stress of RNAi treated animals increased significantly compared to control animals (Figure 6 C, D; p<10^−3^). Conversely, the stress resistance of *smg-1* RNAi animals was reduced by *cep-1* RNAi (Figure 6 C, D; p<10^−3^), as expected from the partial suppression observed for the lifespan phenotype (Figure 6).

Altogether, our results show that CEP-1 has opposing effects on oxidative stress resistance depending on the presence or absence of *smg-1*. This apparent paradox may be explained by the role of p53 in mammalian cells, where it displays either pro-oxidant or anti-oxidant functions depending on the level of oxidative stress (high or low, respectively, [30]. In wild-type animals treated with paraquat, where the level of oxidative stress is high, p53 plays a pro oxidant function, thus explaining the beneficial effect of reducing its expression. Upon *smg-1* inactivation, the levels of oxidative stress may be lower; p53 could play an antioxidant function in this context [30], explaining the deleterious effect of its removal.

Overall, our results show that positive and negative regulators of SMG-1 activity in lifespan regulation act in a similar manner in the oxidative stress response. These data support the hypothesis that the resistance to oxidative stress of *smg-1* animals may be responsible for their increased lifespan phenotype. However, other mechanisms may also be involved since *daf-16* (or *daf-18*) inactivation is sufficient to suppress *smg-1* lifespan regulation without completely inhibiting the *smg-1* stress resistance phenotype.

### A role for SMG-1 in sensory neuron signaling for lifespan regulation

Several observations prompted us to investigate the role of *smg-1* in the regulation of lifespan *via* sensory neurons. Firstly, among the different mechanisms that modulate *C. elegans* lifespan, mutations in sensory neurons lengthen lifespan in a *daf-16* dependent manner [31]. Secondly, a number of genes that act in the nervous system have been shown to be refractory to RNAi in a wild type context, but efficiently inactivated in an *rrf-3* RNAi hyper-sensitive background [17]. Similarly, *smg-1* RNAi increased lifespan in RNAi hyper-sensitive backgrounds such as *rrf-3* and *ppw-1* [32], but not in wild-type animals (Figure 1 and data not shown).

**Figure 7:**
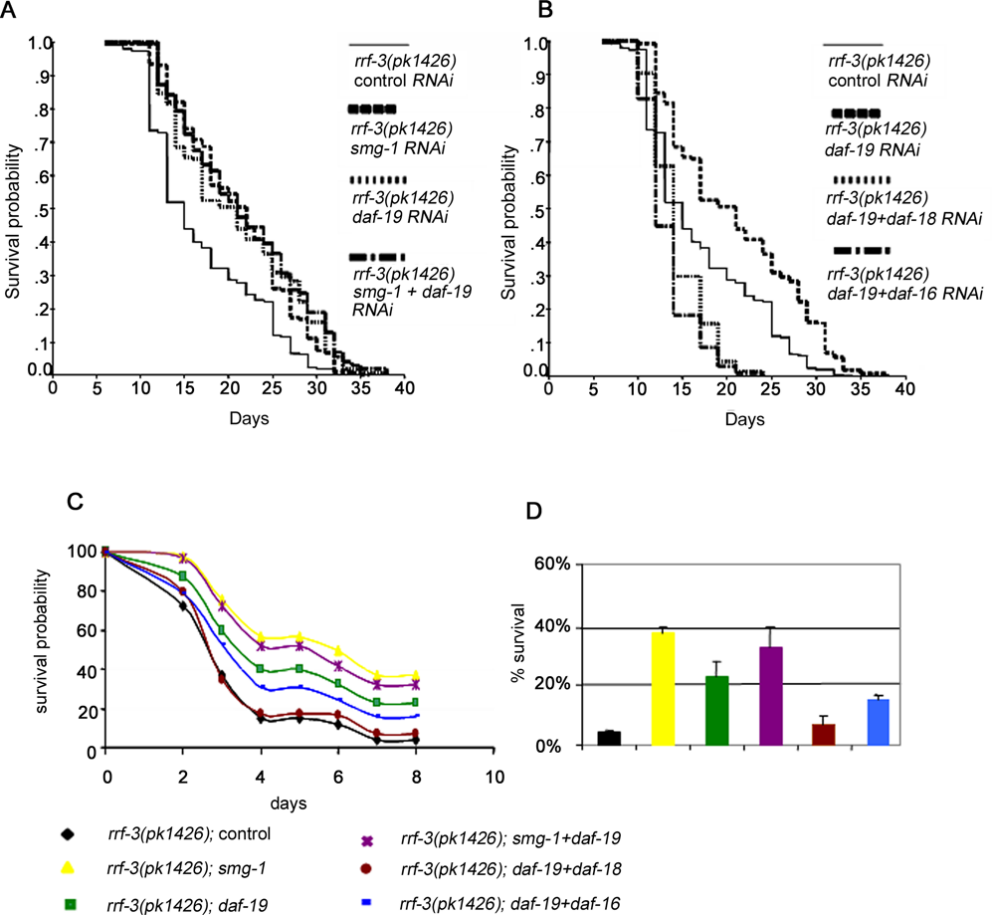
Genetic interaction between *smg-1* and *daf-19* for lifespan and oxidative stress resistance phenotypes. (A, B): Survival curves of *rrf-3(pk1426)* mutants treated with *smg-1* and/or *daf-19* RNAi (A) and treated with *daf-19* RNAi alone or in combination with *daf-18* or *daf-16* RNAi (B). Curves are drawn from data set of Table 1. (C) Survival curves of RNAi treated animals in presence of paraquat (see Materials and Methods). (D) Histograms presented as mean ± SEM at day 7 of paraquat treatment. Genetic interactions for oxidative stress resistance between *daf-19* and either *smg-1*, *daf-18* or *daf-16* are shown.

We therefore asked whether *smg-1* genetically interacts with *tax-4* and *daf-19* , two genes involved in lifespan regulation by sensory neurons [31]. The lifespan of *tax-4(p678); rrf-3(pk1426)* double mutants was increased when these animals were fed with *smg-1* RNAi (Table 1), suggesting that *tax-4* and *smg-1* act independently to regulate lifespan. Conversely, the lifespan of animals fed with both *daf-19* and *smg-1* RNAi was not significantly lengthened compared to either RNAi alone (Table 1; Figure 7A). Furthermore, similarly to *smg-1*, the lifespan increase caused by *daf-19* RNAi inactivation was fully suppressed by *daf-16* RNAi (Table 1; Figure 7B), as previously reported for *daf-16(mu86); daf-19(m86)* double mutants [31], as well as by *daf-18* RNAi (Table 1; Figure 7B).

*daf-19*, which encodes an evolutionary conserved RFX-type transcription factor, is a master gene for the development of a ciliary module in C. *elegans* [33]. In order to assess the role of SMG-1 in sensory neuron formation, animals were stained with DiO, a fluorescent probe which enters through functional cilia (see material and methods).We counted on average 10.2+/−0.2 (n = 50); 9.4+/−0.3 (n = 42); 9.6+/−0.2 (n = 29) and 0 (n = 21) ciliated neurons, respectively, in *rrf-3* animals maintained on control bacteria, *rrf-3* animals on *daf-19* RNAi, in *daf-12(sa204)* mutants, and in *daf-19(m86)*; *daf-12(sa204)* mutants. Therefore, while *daf-19*(*m86)* mutants do not stain at all [33], inhibition of *daf-19* expression by RNAi is not strong enough to interfere with sensory neurons development, as also observed by others (Peter Swoboda, personal communication). Furthermore, *smg-1* RNAi treated animals stained a similar number of sensory neurons as animals fed on control bacteria (10.5+/−0.2, n = 40). Overall our results show that *smg-1* inhibition by RNAi does not compromise sensory neurons formation. These results do not however exclude a possible function for SMG-1 in sensory perception. The *smg-1* predicted promoter contains the canonical DAF-19 target sequence [34], suggesting that *smg-1* expression may be regulated by DAF-19. As *daf-19* expression is restricted to sensory neurons, *smg-1* may well play a role in these cells to regulate lifespan. Nonetheless, the SMG-1 mode of action differs from the previously described pathways, as sensory mutants, but not *smg-1* inactivation, affect DAF-16 nuclear translocation [31].

As observed upon *smg-1* inactivation, resistance to oxidative stress was increased after *daf-19* RNAi, and was suppressed when *daf-19* was inactivated in combination with *daf-16* or *daf-18* (Figure 7C, D). Furthermore, in contrast to *daf-2* and *smg-1* double RNAi, inactivation of *daf-19* did not confer higher resistance to *smg-1* RNAi animals. These results strongly suggest that SMG-1 may function with DAF-19 to regulate both lifespan and oxidative stress.

In conclusion, we identified *smg-1* as a novel gene involved in lifespan regulation. Furthermore, our results suggest that SMG-1 may act independently of DAF-2 and requires DAF-18/PTEN, DAF-16/FOXO and CEP-1/p53 to regulate lifespan.

In mammalian cells, p53 and FOXO3A can act as cofactors to regulate transcription [35]. Furthermore, PTEN has also been shown to interact with and to stabilize the p53 protein [36]. Thus, it is tempting to speculate that SMG-1 may affect DAF-16 transcriptional activity *via* the regulation of PTEN and p53. Interestingly, the physical interaction between p53 and PTEN has recently been shown to be regulated by oxidative stress [37], and their functional crosstalk does not require the lipid phosphatase activity of PTEN [37]. This is in agreement with our results strongly suggesting that SMG-1 acts independently of the PI 3 kinase AGE-1. Finally, pull down experiments revealed that PTEN and hSMG-1 physically interact in human cells [38]. It is therefore possible that mammalian ortholog of *smg-1* control lifespan and the response to oxidative stress in mammals. Recent data suggests a function for SMG-1 both in the oxidative stress response [39] and a role in apoptosis unrelated to the suppression of nonsense-mediated mRNA decay [40]. Understanding the molecular interactions and mechanisms of the pathway involving SMG-1 in aging will be the challenge for future studies.

## Materials and Methods

### Strains

Strains used were as follows: wild-type strains N2 Bristol, daf-18(e1375) IV [13], [41], rrf-3(pk1426) II [17], tax-4(p678) III [42], daf-19(m86) II; daf-12(sa204) X and daf-12(sa204) [33]. smg-1(r861) I [18] and smg-1(tm869), obtained from the C.elegans knockout consortium directed by Pr Mitani, were outcrossed three additional times. The PTCxi strain [24] was kindly provided by D. Longman (J.F. Caceres Lab, MRC, Edinburgh, Scotland).

To construct double mutants *daf-18 (e1375) IV; rrf-3 (pk1426) II*, *rrf-3(pk1426)* males were crossed to *daf-18(e1375)* hermaphrodites and F2 progeny were assayed for sterility at 25°C and incapacity to form dauer on overgrown plates. Double mutants *tax-4(p678) III; rrf-3(pk1426) II* were generated by crossing *tax-4(p678) III* hermaphrodites to *rrf-3(pk1426) II* males. F2 progeny were screened for sterility at 25°C and dauer constitutive formation at 27°C. To obtain single mutants *unc-54(r293)* I, N2 males were mated to *smg-3(r930) IV; unc-54(r293) I* and paralyzed F2 were isolated. To generate *rrf-3(pk1426)* mutants carrying the DAF-16∶∶GFP transgene, *rrf-3(pk1426)* males were crossed to TJ356 [6] hermaphrodites. F2 rollers were assayed for sterility at 25°C.

### RNAi experiments

Bacterial feeding RNAi experiments were carried out essentially as described previously [43]. Briefly, single colonies of HT115 bacteria containing plasmids of interest were first grown overnight in LB with 100 µg/ml ampicillin and 12.5 µg/ml tetracyclin and then for 8 h in LB with 100 µg/ml ampicillin. Bacteria were seeded directly onto NGM plates containing 2 mM IPTG and 25 µg/ml carbenicillin. Clones used: C48B6.6 and C48B6.7 (*smg-1*), F46B6.3 (*smg-4*), W02D3.8 (*smg-5*), Y43B6A.a (*smg-7*), B0334.8 (*age-1*), C12D8.10 (*akt-1*), R13H8.1 (*daf-16*), F52B5.4 (*cep-1*), F33H1.1 (*daf-19*) and T07A9.6 (*daf-18*) have been purchased from. *daf-2* and *smg-2* clones were kindly provided by C. Kenyon lab (University of California, San Francisco, USA) and D. Longman (J.F. Caceres Lab, MRC, Edinburgh, Scotland) respectively. Each clone has been sequenced to confirm its identity.

Double RNAi experiments were carried out by mixing the bacterial cultures directly before seeding the NGM plates. Controls were RNAi clone 50% diluted with vector control RNAi bacteria.

### Lifespan assays

Animals were grown on regular NGM plates at 20°C until reaching the L4 stage and then transferred to RNAi plates (F0). F0 adults were removed after 24h and F1 L4 were transferred to 10 µM 5-fluorodeoxyuracile (5-FU, Sigma-Aldrich, Steinheim, Germany) containing plates to prevent growth of progeny. Lifespan assays were performed at 20°C. The day of the shift is counted as day 0 in the adult lifespan assay. Control and experimental animals were transferred in parallel to fresh RNAi plates once a week. Lifespan was assessed every 2–3 days and animals were scored as dead when they ceased moving and responding to prodding. Animals that crawled off the plate, had a “protruding vulva” or an “exploded vulva” phenotype were censored. *smg-1* RNAi was also performed in absence of 5-FU and gave similar results (data not shown).

Survival analyses were performed using the Kaplan Meier method and the significance of differences between survival curves calculated using the log rank test. The statistical software used was SPSS, Version 11.5 (SPSS, Chicago, IL, USA) and all P-values<0.05 were considered significant.

### Assessment of NMD activity in living worms

Animals carrying the PTC transgenic reporter [24] were fed at 20°C with the control clone only or with the *smg-1* RNAi clone mixed either with the control, or *daf-18*, or *daf-16* RNAi clones. F1 animals were scored for GFP expression at the L4 stage. For GFP intensity quantification, animals were photographed under a GFP filter and the average brightness was determined for each photograph by Lucia Nikon software. All images were handled identically. At least 30 animals per RNAi conditions were averaged.

### Stress resistance assays

Synchronously cultured animals were kept on NGM plates at 20°C until the young adult stage. For each strain, 5 × 20 young adults were transferred on Paraquat (methylviologene, Sigma-Aldrich, Steinheim, Germany) containing plates (90 µl of 150 mM Paraquat added on top of NGM plates already seeded with HT115 bacteria). Surviving animals were scored every day during 8–9 days. At least three independent RNAi experiments have been conducted for each clone tested. *P*-values were calculated using the t-Student test to determine differences in oxidative stress resistance.

### Larval arrest assays

Five young adults *daf-2(e1370) ; rrf-3(pk1426)* double mutants were fed at 22°C with either HT115 or *smg-1* RNAi bacteria, then removed 24 hours later. F1 progenies were followed every day and the numbers of worms that have reached the adult stage were counted at day 4. Numbers are given for 3 independent experiments.

### Observation of DAF-16∶∶GFP sub-cellular localization and DiO staining

The sub-cellular localization of the DAF-16∶∶GFP protein was analyzed in the *smg-1(r861)* mutant background and after *smg-1* RNAi inactivation in a *rrf-3(pk1426)* mutant background by fluorescence microscopy under a GFP filter. About 10 worms were mounted on agar pads (2% agarose with 5 mM tetramisole) to avoid DAF-16∶∶GFP translocation due to stress [8]. At least 20 animals were examined for each developmental stage (embryo, L1, L2, L3, L4 and adult).

Sensory neurons were stained by incubating L4 worms in M9 containing DiO (Molecular Probes) at 10 µg/ml final concentration for two hours. Worms were then transfered to plates for one hour and observed by fluorescence microscopy under a GFP filter.

## Supporting Information

Table S1 List of clones tested in the screen.
(0.03 MB XLS)

Table S2 Lifespan data for individual experiments
(0.33 MB DOC)
