# Correction: A Novel Role for the SMG-1 Kinase in Lifespan and Oxidative Stress Resistance in *Caenorhabditis elegans*

**DOI:** 10.1371/annotation/8b010ad6-34d5-45fc-9bc2-c82a7370ebc7

**Published:** 2009-01-14

**Authors:** Ingrid Masse, Laurent Molin, Laurent Mouchiroud, Philippe Vanhems, Francesca Palladino, Marc Billaud, Florence Solari

The allele name tm869 appears incorrectly throughout the manuscript. The correct allele name should be tm849.

