# Supplementary material for: A Novel Role for the SMG-1 Kinase in Lifespan and Oxidative Stress Resistance in Caenorhabditis elegans
Source: PLoS One. 2008 Oct 6;3(10):e3354. doi: 10.1371/journal.pone.0003354 (PMC2556085; doi:10.1371/journal.pone.0003354)
Supplement: Table S2 — Lifespan data for individual experiments (0.33 MB DOC) [file pone.0003354.s002.doc]

| **Genotype** | **RNAi** | **Mean**  **lifespan** | **SEM** | **n** | ***p value against control** |
| --- | --- | --- | --- | --- | --- |
| **wild-type** |  | 17.2 | 0.505 | 95 |  |
| ***smg-1(tm869)*** |  | 18.1 | 0.993 | 24 | 0.958 |
|  |  |  |  |  |  |
| ***Wild-type*** |  | 14.5 | 0.654 | 66 |  |
| ***smg-1(tm869)*** |  | 19.6 | 0.545 | 74 | <10^-3^ |
|  |  |  |  |  |  |
| ***Wild-type*** |  | 16.7 | 0.749 | 68 |  |
| ***smg-1(tm869)*** |  | 20 .4 | 0.492 | 89 | <10^-3^ |
|  |  |  |  |  |  |
| ***rrf-3(pk1426)*** | **control** | 15.2 | 0.944 | 44 |  |
|  | **smg-1** | 21.8 | 1.056 | 37 | <10^-3^ |
|  |  |  |  |  |  |
| ***rrf-3(pk1426)*** | **control** | 15.2 | 0.448 | 113 |  |
|  | **smg-1** | 17.8 | 0.552 | 94 | <10^-3^ |
|  |  |  |  |  |  |
| ***rrf-3(pk1426)*** | **control** | 19 | 0,857 | 59 |  |
|  | **smg-1** | 24,3 | 0,953 | 50 | <10^-3^ |
|  |  |  |  |  |  |
| ***rrf-3(pk1426);***  ***daf-18(e1375)*** | **control** | 14,1 | 0,370 | 60 |  |
|  | **smg-1** | 14,7 | 0,474 | 29 | 0,502 |
|  |  |  |  |  |  |
| ***rrf-3(pk1426);***  ***daf-18(e1375)*** | **control** | 13.3 | 0.350 | 63 |  |
|  | **smg-1** | 13.9 | 0.518 | 0.176 |  |
|  |  |  |  |  |  |
| ***rrf-3(pk1426);***  ***daf-18(e1375)*** | **control** | 11 | 0,260 | 79 |  |
|  | **smg-1** | 11 | 0,258 | 74 | 0,936 |
|  |  |  |  |  |  |
| ***rrf-3(pk1426);***  ***daf-18(mg198)*** | **control** | 11,3 | 0,312 | 42 |  |
|  | **smg-1** | 11,2 | 0,430 | 22 | 0.653 |
|  |  |  |  |  |  |
| ***rrf-3(pk1426)*** | **control** | 17,6 | 0,800 | 51 |  |
|  | **smg-1** | 22,9 | 0,751 | 49 | <10^-3^ |
|  |  |  |  |  |  |
| ***rrf-3(pk1426);***  ***daf-18(mg198)*** | **control** | 11,4 | 0,731 | 14 |  |
|  | **smg-1** | 10,3 | 0,517 | 10 | 0,321 |
|  |  |  |  |  |  |
| ***rrf-3(pk1426)*** | **control** | 17.2 | 0.685 | 76 |  |
|  | **smg-1** | 23.8 | 0.729 | 52 | <10^-3^ |
|  |  |  |  |  |  |
| ***rrf-3(pk1426);***  ***daf-18(mg198)*** | **control** | 10.4 | 0.349 | 57 |  |
|  | **smg-1** | 10.6 | 0.336 | 74 | 0.554 |
|  |  |  |  |  |  |
| ***rrf-3(pk1426)*** | **control** | 17,2 | 0,713 | 74 |  |
|  | **smg-1** | 19,9 | 1,047 | 42 | 0,026 |
|  |  |  |  |  |  |
|  | **akt-1 control** | 24,5 | 0,913 | 58 |  |
|  | **akt-1;smg-1** | 30,1 | 0,761 | 81 | <10^-3^ |
|  |  |  |  |  |  |
|  | **daf-16 control** | 12,1 | 0,175 | 45 |  |
|  | **daf-16;smg-1** | 12,1 | 0,203 | 49 | 0,884 |
|  |  |  |  |  |  |
| ***rrf-3(pk1426)*** | **control** | 17,1 | 0,694 | 87 |  |
|  | **cep-1** | 18,7 | 0,801 | 79 | 0,187 |
|  |  |  |  |  |  |
|  | **smg-1 control** | 24 | 0,726 | 79 |  |
|  | **smg-1;cep-1** | 21 | 0,839 | 69 | 0,010 |
|  |  |  |  |  |  |
|  | **daf-2 control** | 32,1 | 0,963 | 89 |  |
|  | **smg-1;daf-2** | 36,1 | 0,946 | 89 | 0,002 |
|  |  |  |  |  |  |
|  | **age-1 control** | 22,9 | 1,121 | 86 |  |
|  | **age-1;smg-1** | 31,7 | 1,154 | 78 | <10^-3^ |
|  |  |  |  |  |  |
| ***rrf-3(pk1426)*** | **control** | 19,2 | 0,767 | 77 |  |
|  | **smg-1** | 22,2 | 0,796 | 61 | 0,002 |
|  |  |  |  |  |  |
|  | **daf-2 control** | 33,5 | 1,086 | 62 |  |
|  | **smg-1;daf-2** | 39,1 | 0,742 | 58 | <10^-3^ |
|  |  |  |  |  |  |
|  | **age-1 control** | 26,4 | 0,998 | 84 |  |
|  | **age-1;smg-1** | 30,5 | 1,043 | 69 | 0,005 |
|  |  |  |  |  |  |
|  | **akt-1 control** | 25,9 | 0,885 | 80 |  |
|  | **akt-1;smg-1** | 29,1 | 0,834 | 53 | 0,093 |
|  |  |  |  |  |  |
|  | **daf-16 control** | 12,6 | 0,329 | 69 |  |
|  | **daf-16;smg-1** | 12,5 | 0,348 | 65 | 0,867 |
|  |  |  |  |  |  |
| ***rrf-3(pk1426)*** | **control** | 17,4 | 0,775 | 78 |  |
|  | **smg-1** | 22 | 1,095 | 47 | <10^-3^ |
|  | **smg-5** | 18,6 | 0,690 | 78 | 0,442 |
|  | **smg-4** | 18,7 | 0,707 | 67 | 0,416 |
|  | **smg-7** | 16,9 | 0,753 | 69 | 0,810 |
|  |  |  |  |  |  |
| ***rrf-3(pk1426);tax-4(p678)*** | **control** | 19,8 | 0,702 | 72 | 0,030 |
|  | **smg-1** | 24,9 | 0,971 | 52 | <10^-3^ |
|  |  |  |  |  |  |
| ***rrf-3(pk1426)*** | **control** | 16,8 | 0,824 | 72 |  |
|  | **smg-1(C48B6.7)** | 20,7 | 0,959 | 53 | 0,007 |
|  | **smg-1(C48B6.6)** | 20,9 | 0,856 | 68 | 0,003 |
|  |  |  |  |  |  |
|  | ****smg-1 control** | 20,9 | 0,636 | 121 |  |
|  | **cep-1** | 18,1 | 0,718 | 79 | 0,610 |
|  | **smg-1;cep-1** | 19,3 | 0,708 | 83 | 0,082 |
|  |  |  |  |  |  |
|  | **daf-19 control** | 20,1 | 0,919 | 58 |  |
|  | **daf-19;smg-1** | 20,4 | 0,977 | 57 | 0,442 |
|  |  |  |  |  |  |
|  | **daf-2 control** | 30,4 | 1,359 | 68 |  |
|  | **age-1** | 23,2 | 0,986 | 78 |  |
|  | **daf-2;age-1** | 30,2 | 1,142 | 86 | 0,671 |
|  |  |  |  |  |  |
| ***rrf-3(pk1426)*** | **daf-2 control** | 28,2 | 1,191 | 70 |  |
|  | **age-1** | 21,4 | 1,140 | 65 |  |
|  | **daf-2;age-1** | 29,8 | 1,168 | 74 | 0,425 |
|  |  |  |  |  |  |
| ***rrf-3(pk1426)*** | **control** | 16,6 | 0,696 | 79 |  |
|  | **smg-1** | 20 | 0,792 | 56 | 0,010 |
|  |  |  |  |  |  |
| ***rrf-3(pk1426)*** | **control** | 17,5 | 0,887 | 72 |  |
|  | **smg-1** | 22,8 | 0,739 | 82 | 0,002 |
|  | **cep-1** | 19,1 | 0,792 | 87 | 0,661 |
|  |  |  |  |  |  |
|  | **smg-1 control** | 22,8 | 0,739 | 82 |  |
|  | **smg-1;cep-1** | 19,9 | 0,777 | 75 | 0,012 |
|  |  |  |  |  |  |
|  | **smg-1 control** | 22,8 | 0,739 | 82 |  |
|  | **daf-19** | 19,7 | 0,983 | 57 |  |
|  | **smg-1;daf-19** | 22,6 | 0,790 | 72 | 0,851 |
|  |  |  |  |  |  |
|  | **daf-18 control** | 14,8 | 0,418 | 82 |  |
|  | **daf-19;daf-18** | 14,1 | 0,455 | 65 | 0,290 |
|  |  |  |  |  |  |
|  | **daf-16 control** | 14,1 | 0,325 | 76 | 0,001 |
|  | **daf-19;daf-16** | 14,1 | 0,429 | 61 | 0,804 |
|  |  |  |  |  |  |
| ***rrf-3(pk1426)*** | **control** | 18,7 | 0,688 | 74 |  |
|  | **smg-1** | 24,3 | 0,657 | 75 | < 0,0001 |
|  |  |  |  |  |  |
|  | **smg-1 control** | 24,3 | 0,657 | 75 |  |
|  | **daf-19** | 23,5 | 1,051 | 47 |  |
|  | **daf-19;smg-1** | 23,8 | 1,061 | 44 | 0,838 |
|  |  |  |  |  |  |
|  | **daf-18 control** | 14,5 | 0,307 | 62 |  |
|  | **daf-19;daf-18** | 14,7 | 0,293 | 69 | 0,500 |
|  |  |  |  |  |  |
|  | **daf-16 control** | 13,2 | 0,395 | 42 |  |
|  | **daf-19;daf-16** | 12,3 | 0,215 | 44 | 0,062 |
|  |  |  |  |  |  |
| ***rrf-3(pk1426);tax-4(p678)*** | **control** | 25,3 | 0,644 | 74 |  |
|  | **smg-1** | 27,9 | 0,737 | 53 | 0,012 |
|  |  |  |  |  |  |
| ***rrf-3(pk1426)*** | **control** | 15,4 | 0,509 | 86 |  |
|  | **smg-1** | 18,2 | 0,617 | 53 | 0,006 |
|  | **smg-2** | 15,5 | 0,546 | 82 | 0,907 |
|  |  |  |  |  |  |
| ***rrf-3(pk1426)*** | **control** | 16,7 | 0,676 | 71 |  |
|  | **smg-1** | 21,4 | 0,594 | 61 | <10^-3^ |
|  | **smg-2** | 16,4 | 0,647 | 76 | 0,852 |
|  |  |  |  |  |  |
| ***rrf-3(pk1426)*** | **control** | 18,6 | 0,925 | 65 |  |
|  | **smg-5** | 18,4 | 0,648 | 55 | 0,353 |
|  |  |  |  |  |  |
| ***rrf-3(pk1426)*** | **control** | 18,3 | 0,876 | 61 |  |
|  | **smg-4** | 19,1 | 1,196 | 27 | 0,955 |
|  | **smg-7** | 18,9 | 1,419 | 22 | 0,725 |

**Table S2: Lifespan analysis for individual experiments.**

Individual experiments are separated by a grey line. All experiments were carried out by RNAi feeding at 20°C. **P*-values from a Log rank test were calculated by pair-wise comparisons to the control (no RNAi insert or specific group as indicated for each experiment). *P-*values less than 0,05 are considered statistically significant, demonstrating that the two lifespan populations are different.** results obtained with two independent RNAi feeding clones that gave similar results have been pooled for comparisons.
